# Application of the Left Ventricular Pressure–Strain Loop Technique in Monitoring Improvement Factors of Patients With Heart Failure Reduced Ejection Fraction

**DOI:** 10.1155/cdr/5562513

**Published:** 2024-12-17

**Authors:** Qing Li, Yaolei Guo, Xiaomin Tang, Chang Liu, Zhidong Wang, Qianping Gao, Yuanshi Li, Junxian Cao

**Affiliations:** ^1^Second Department of Cardiology, Binzhou People's Hospital, Binzhou 256600, China; ^2^Department of Echocardiography, The First Affiliated Hospital of Harbin Medical University, Harbin 150001, China; ^3^Department of Hematopathology, The First Affiliated Hospital of Jinzhou Medical University, Jinzhou 121012, China; ^4^Department of Cardiology, The First Affiliated Hospital of Harbin Medical University, Harbin 150001, China

**Keywords:** echocardiography, heart failure, left ventricular ejection fraction, myocardial work, pressure–strain loop

## Abstract

The left ventricular pressure–strain loop (PSL) is a new technique based on ultrasound for noninvasive quantitative evaluation of global and local myocardial work (MW). This study is aimed at evaluating improvement factors of patients with heart failure (HF) reduced ejection fraction (HFrEF) using the PSL technique. A total of 88 patients with HF were enrolled in this study, which had ≤ 40% left ventricular ejection fraction (LVEF). The EchoPAC workstation was used to obtain the global longitudinal strain (GLS) and MW parameters of the left ventricle. All patients have taken medicines for HF treatments for 6–12 months. The improvements of HF after therapies were evaluated according to the following recommended criteria. The clinical characteristics of patients with improved and nonimproved groups were stratified via univariate or multivariate logistic regression analysis, receiver operating characteristic (ROC), and the area under ROC (area under the curve (AUC)). There were no significant differences in general medical information, the underlying diseases, laboratory findings, myocardial enzyme activities, and taking medicines between the improved and nonimproved LVEF patients (*p* > 0.05). There were significant differences in LVEF of patients at admission, left ventricular end-diastolic diameter (LVEDD), interventricular septum thickness (IVST), early diastolic mitral flow peak velocity E (E peak), GLS, global myocardial work index (GWI), global myocardial constructive work (GCW), and global myocardial work efficiency (GWE) between the two groups (*p* < 0.05). Univariate and multivariate logistic regression analyses confirmed that GWI and GCW were critical predictive factors for LVEF improvement in patients with HF. ROC curve showed that the AUC of GWI and GCW were 0.796 and 0.779 at the cut-off of 741 mmHg% for GWI and 973.5 mmHg% for GCW, respectively. The sensitivities of GWI and GCW were 65% and 75%, and the specificities of GWI and GCW were 83.3% and 79.2% at given cut-off values. These results revealed that GWI and GCW were independent predictors of improvement of LVEF in patients with HFrEF.

## 1. Introduction

Heart failure (HF) is a disease status at late stages from various cardiac diseases. Globally, HF is annually involved in more than 60 million patients with high morbidity and mortality [1]. Most patients with HF have inferior quality of life and cost expensive health burden. Therefore, the exact evaluation of healthy conditions in patients with HF is no doubt critical for the improvement of HF. In April 2022, the American Heart Association (AHA) proposed a new guideline for HF diagnostic criteria [2]: (i) Heart failure reduced ejection fraction (HFrEF) is left ventricular ejection fraction (LVEF) ≤ 40%; (ii) heart failure with midrange ejection fraction (HFmrEF) is 41% ≤ LVEF ≤ 49%; (iii) heart failure with preserved ejection fraction (HFpEF) is LVEF ≥ 50%; and (iv) heart failure with improved ejection fraction (HFimpEF) is a baseline LVEF ≤ 40%; LVEF increases 10% from baseline or LVEF > 40% after treatments. In China, clinical diagnosis for HF is similar to AHA criteria [3]. Therefore, all physicians can accurately evaluate the improvement status of HF patients with therapies following these new criteria.

The two-dimensional speckle tracking echocardiography (2D-STE) is sensitive and reliable equipment for HF examination and measurement of the left ventricular systolic functions [4]. The left ventricular pressure–strain loop (PSL) detected by 2D-STE is a new technique for noninvasive quantification of global and local myocardial work (MW), which has great potential value in evaluating the severity, early diagnosis, and prognosis of patients with HF [5–7]. PSL can measure myocardial strain, pressure load, global longitudinal strain (GLS), and peak pressure of the left ventricle (LV) using a two-dimension area as MW [8], which is obtained via calculating the 17-segment MW index model combined time course of the LV and myocardial contractility [9, 10]. PSL can also input the following critical parameters reflecting LV systolic functions: global myocardial work index (GWI), global myocardial constructive work (GCW), global myocardial wasted work (GWW), and global myocardial work efficiency (GWE) [11]. Among these MW parameters, GWI is strongly relative to peak oxygen consumption and N-terminal protein hormone B-natriuretic peptide (NT-proBNP) level [12]; GCW reflects the energy consumption in LV ejection [13]; GWW is associated with the synchrony of LV myocardial contraction [5]; GWE is relevant to the net percentage of total MW that contribute to cardiac output (CO) [14], respectively. Compared to other MW parameters, GCW may be perceived as superior because GCW provides a comprehensive assessment of the overall myocardial functions by considering both the systolic and diastolic phases of the cardiac cycle. In addition, GCW is also superior to the other three parameters in reflecting global perspective, clinical relevance, and sensitivity to changes for myocardial functions [15, 16]. Therefore, the extensive advantages of PSL drive this technique to be applied in many studies for cardiac functions assessment of HF, hypertension (HTN), and Type2 diabetes mellitus (DM) [17, 18], including evaluation of the LV functions, early warning of cardiac dysfunctions, identification of the severe cases, and prediction of long-term outcomes [19–21]. In addition, LVEF and PSL combinative measurements more accurately detect cardiac functions than LVEF assessment alone in chemotherapies of cancer patients [22, 23]. The study demonstrated that LVEFs were normal, but PSL and myocardial work indices (MWIs) were significantly reduced at 3 and 6 months of measurement after chemotherapies in patients with breast cancer [23], indicating PSL technique is a more accurate and sensitive tool for evaluating cardiac functions.

Recent studies have revealed that MW parameters are closely related to the prognosis of patients with HF caused by various diseases and the improvement of LVEF [24–26]. However, the exact independent factors for predicting improvements of HFrEF patients were unclear. This study is aimed at investigating the noninvasive evaluation of MW in patients with HFrEF by PSL and at exploring the predictive value of MW parameters for improvements of LVEF in HFrEF patients.

## 2. Materials and Methods

### 2.1. Patients

To assess critical factors for the improvement of HF after therapies, a prospective study was designed. A total of 88 patients were enrolled in this study, who were diagnosed as HF with LVEF ≤ 40% and admitted to the Department of Cardiology of the First Affiliated Hospital of Harbin Medical University from September 2021 to August 2022. The patients were recruited according to the following criteria: (i) HF was diagnosed based on the “Chinese Heart Failure Diagnosis and Treatment Guidance” [3]; (ii) LVEF was ≤ 40% according to echocardiography examination; (iii) medical information must be complete, which had not lost medical records, including medical histories, laboratory findings, and echocardiograph data; (iv) 2D-STE images of patient were available; (v) all patients compliance were good following medicine use for 6–12 months after they were discharged from hospital. The enrolled patients were diagnosed as acute decompensated HF or acute decompensation of chronic HF. The exclusion criteria are as follows: (i) the patients with pacemakers or implantable cardioverter–defibrillators (ICDs), which enhance recovery of myocardial contractility, but affect the collection of PSL image precision; (ii) cardiac arrhythmia including atrial fibrillation or flutter, left bundle branch block, frequent premature ventricular contractions, and irregular R–R intervals; (iii) severe primary valvular heart disease or congenital heart disease; (iv) obstructive hypertrophic cardiomyopathy; (v) occlusive peripheral vascular disease; (vi) abnormal liver functions (ALT or aspartate transferase (AST) > 3 upper limit of normal (ULN) and kidney functions (estimated glomerular filtration rate (eGFR) < 30 mL/min/1.73m^2^); (vii) poor ultrasound images; and (viii) incomplete medical information. This study was reviewed and approved by the ethical committee of the First Affiliated Hospital of Harbin Medical University (Approval #: 2023XJSS96, Harbin City, China). Written informed consent was obtained before the study from all patients.

### 2.2. Participant Enrollment and Follow-Up

LVEF improvement criteria were determined according to the AHA proposed of a new guideline for HF diagnostic criteria in April 2022 [2] and the *Journal of the American College of Cardiology* (*JACC*) Expert Consensus in 2020 [27]: HFrEF is defined as LVEF is ≤ 40% baseline, LVEF increase > 10% from LVEF baseline or > 40% after treatments. Then, all patients were followed up at 6, 9, 12 months after discharge and performed echocardiography examination. These patients were further divided into two groups according to twice follow-up LVEF values: (1) improved LVEF group: Baseline LVEF was ≤ 40%; twice follow-up showed that LVEF was > 40% and increased ≥ 10% from baseline LVEF after treatments; (2) nonimproved LVEF group: Baseline LVEF was ≤ 40%; LVEF was still ≤ 40% or increased < 10% from baseline after treatments.

### 2.3. Clinical and Laboratory Data Collection

After patients were admitted to the hospital, their basic medical data were recorded, including age, gender, systolic blood pressure (SBP), diastolic blood pressure (DBP), body height, body weight, heart rate (HR), the underlying diseases, smoking or drinking histories, and body mass index (BMI). Laboratory tests included hemoglobin (Hb), HbA1C, platelet count, creatinine, blood lipid, blood potassium, albumin (ALB), troponin I (TNI), creatine kinase isoenzyme MB (CK-MB), and N-terminal probrain natriuretic peptide (NT-proBNP) levels. Medicine uses included the following alone or a combination of five kinds of drug: (1) angiotensin receptor–neprilysin inhibitor (ARNI)/angiotensin-converting enzyme inhibitor (ACEI)/angiotensin receptor blocker (ARB). Briefly, ARNI: Entresto, 25–200 mg, oral, bid; ACEI: enalapril, 5–20 mg, oral, qd; ARB: valsartan, 80–160 mg, oral, qd. If SBP was < 95 mmHg or serum potassium was > 5.5 mmol/L, drugs had been stopped; (2) *β*-receptor blockers: metoprolol succinate, 47.5–190 mg, oral, qd, or bisoprolol, 2.5–5 mg, oral, qd. If SBP was < 95 mmHg or HR was less than 60 beats per minute (BPM), drugs had been stopped; (3) aldosterone receptor antagonist (MRA): spironolactone, 20 mg, oral, qd; (4) SGLT-2 receptor inhibitor: dapagliflozin, 10 mg, oral, qd, or empagliflozin, 10 mg, oral, qd; (5) diuretics: furosemide, 20–100 mg, iv, qd, or torasemide, 20–100 mg, iv, qd, during hospitalization; furosemide, 20–60 mg, oral, qd, or torasemide, 20–40 mg, oral, qd, after patients were discharged from hospital. All drugs were given at the maximal tolerated dose or recommended maximal dose for all follow-up times except for some contraindications.

### 2.4. Echocardiography Image Collection

To accurately assess the patient's heart status, a GE Vivid E95 Color Doppler Ultrasound Machine equipped with an EchoPAC203 workstation and M5sc probe (frequency 2.0–4.5 MHz) was used for GLS and MW parameters analysis. This color pulsed wave Doppler echocardiography was used to determine the left ventricular posterior wall thickness (LVPWT), interventricular septum thickness (IVST), and left ventricular end-diastolic diameter (LVEDD) using the biplane Simpson method. Peak early (E) of the mitral flow velocity/peak early diastolic mitral annular velocity (e⁣′) ratio (E/e⁣′) was measured and calculated via a pulse regular and tissue Doppler in the apical (AP) four-chamber view (CV). Echocardiography images were taken at least twice in each patient. The first echocardiography images were obtained in 48 h after admission; the second echocardiography images were obtained in 6–12 months after discharge from the hospital. All PSL images were taken by the same operator in our hospital. To minimize echocardiography laboratory variation in assessing LVEF, all echocardiography images and MW parameters calculation followed the same standardized protocol and the same machine operator. Color Doppler ultrasound machines were also maintained at regular time. The hospitalization time of all patients was 3–17 days range. The improvement of heart functions was monitored by echocardiography at 6 and 12 months after patients were discharged from the hospital because LVEF improvements take longer time.

### 2.5. MW Analysis

**Figure 1:**
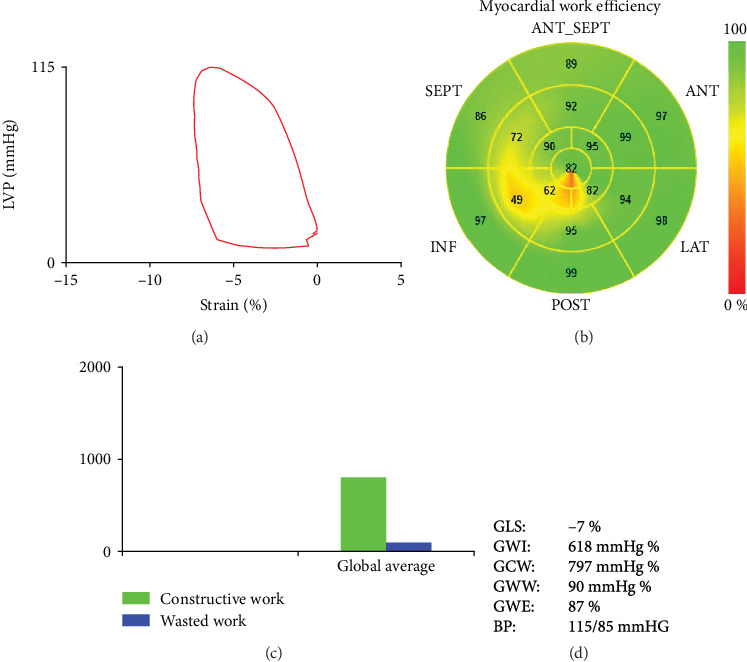
PSL technique shows the left ventricle myocardial work. This image reveals the left ventricle myocardial work parameters by echocardiography. (a) PSL curve, (b) the 17-segment bull's-eye MW plots, (c) the comparison of GCW (green color) and GWW (blue color), and (d) the parameter reports of MW. PSL, pressure–strain loop; MW, myocardial work; GWI, global myocardial work index; GCW, global constructive work; GWW, global wasted work; GWE, global work efficiency; BP, blood pressure.

**Figure 2:**
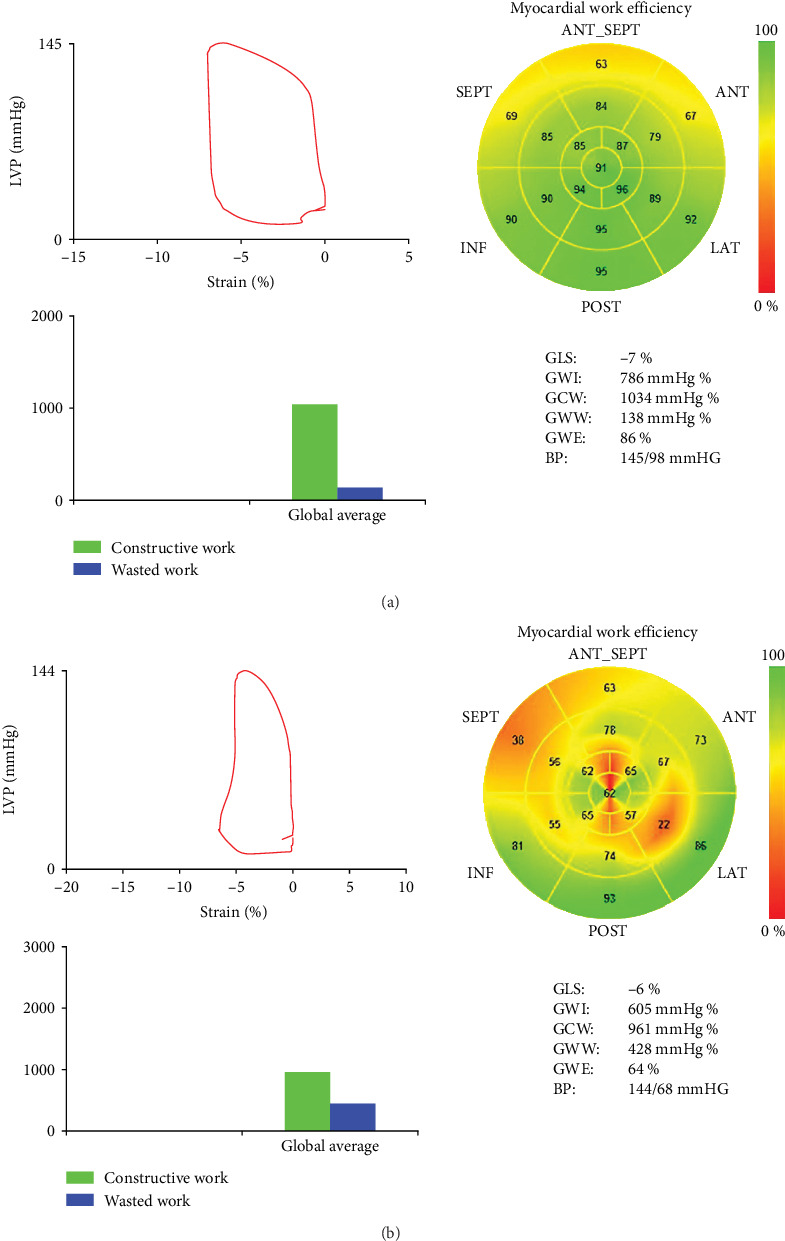
PSL and myocardial work parameters in improved and nonimproved LVEF patients. (a) PSL image in improved LVEF patients; (b) PLS image in nonimproved LVEF patients. PSL, pressure–strain loop; MW, myocardial work; GWI, global myocardial work index; GCW, global constructive work; GWW, global wasted work; GWE, global work efficiency; BP, blood pressure.

The probe was placed at the left ventricular AP of patients, connecting with a 2-dimensional (2D) echocardiography machine. Then, 4-, 3-, and 2-CV images were recorded for five cardiac cycles and stored on a flash driver for further analysis. According to the 2D strain analysis mode, open and close time of mitral and aortic valves, we finally obtained the global GLS, GWI, GCW, GWW, and GWE data as shown in Figure 1. PSL comparison of improved and nonimproved LVEF patients is shown in Figure 2.

### 2.6. Statistical Analysis

We analyzed all data using the SPSS software (25 version, IBM Corp, United States). The normal distribution of data was determined by the Kolmogorov–Smirnov method. The data of continuous variables are present as mean ± standard deviation (SD) (mean ± SD) using *T* test comparison between two groups. If data was not normal distribution, they present as median and interquartile range (P25, P75). The differential comparison was performed using nonparametric rank sum test. Categorical variables were presented as frequency or percentage and compared by the *χ*^2^ test among the groups. Univariate logistic regression model was used for analyzing the effective factors for LVEF improvement. If there were statistical differences in the univariate regression model, we further performed a multivariate logistic regression model. For the predictive value of MW in LVEF improvement, we generated the receiver operating characteristic (ROC) curve and calculated the area under the curve (AUC). The associations between MW parameters and LVEF improvement were analyzed using the Spearman correlation coefficients. *p* value < 0.05 means statistical differences.

## 3. Results

### 3.1. Clinical and Echocardiography Characteristics

To get the final clinical features of patients, 116 HF patients with LVEF ≤ 40% in our hospital were initially selected as study subjects and were given medicine therapies from September 2021 to August 2022. After follow-up to 6–12 months, 28 cases were lost, including 22 cases without regular examination or oral drugs and six dead cases. The remaining 88 patients enrolled in this study. The clinical characteristics of patients with improved and nonimproved LVEF groups were compared (Table 1), including age, sex, basic vital signs, medical history, the New York Heart Association (NYHA class) at admission, smoking or alcohol consumption, platelet, Hb, HbA1C, and CK-MB levels. In the entire study cohort, male and female patients in the 29–85 years old range were 58 cases (58/88, 66%) and 30 cases (30/88, 34%), respectively. During follow-up time with echocardiography examination, 40 patients have experienced LVEF improved from HFrEF to heart failure with recovered ejection fraction (HFrecEF), which accounted for 45.5% of the entire patients. The remaining 48 patients still had nonimproved LVEF (54.5%) after medicine use. The patients with the improved LVEF group included 35% DM, 65% HTN, 10% coronary artery disease (CAD), and 17.5% arrhythmia. The NYHA class at admission in the improved LVEF group was 2.5% Class I, 7.5% Class II, 30.0% Class III, and 60.0% Class IV. There were no significant differences in age, medical history, NYHA class, smoky and drinking histories, SBP, DBP, HR, BW, BMI, platelet, Hb, HbA1C, NT-proBNP, ALB, potassium, TNI, and CK-MB.

We also evaluated the effects of medicine on LVEF of patients, including ARNI/ACEI)/ARB, *β* receptor blocker; MRA, SGLT-2 receptor inhibitor, and diuretics. Results showed that there were no significant LVEF differences between improved and nonimproved groups using various medicines (Table S1).

### 3.2. Comparison of Conventional LV Echocardiography Parameters in Improved and Nonimproved LVEF Groups

To explore the LV functions in improved and nonimproved LVEF patients, we compared their LVEF, LVEDD, IVST, LVPWT, early diastolic mitral flow peak velocity E (E peak), E/e', GLS, GWI, GCW, GWE, and GWW. The results are shown in Table 2 and Figure 1. The MW-relevant parameters, including GWI, GCW, GWE, and GWW, are presented in Figure 1 by 2D-STE. Then, we compared these parameter differences in improved and nonimproved LVEF groups. The results showed that there were significant differences (*p* < 0.05) in LVEF, LVEDD, IVST, E peak, GLS, GWI, GCW, and GWE of the two groups. In contrast, LVPWT, E/e⁣′, and GWW at admission in two group patients had no dramatic differences (*p* > 0.05).

We further analyzed the correlations between the MW parameter and improved LVEF patients. The results indicated that GWI, GCW, and GWE were significantly associated with the LVEF improvement of patients except for GWW (Table 3, *p* < 0.05).

### 3.3. Univariate and Multivariate Logistic Regression Analysis in Patients With the Improved LVEF Group

To further investigate contribution factors for the improved LVEF group, we firstly performed univariate logistic regression analysis using 23 variables, including sex, age, drinking history, BMI, Hb, TNI, CK-MB, blood sugar, NT-proBNP, LVEF, IVST, LVEDD, LVPWT, E peak, E/e⁣′, GLS, GWI, GWW, GWE, GCW, HbA1c, platelet count, and hospitalization time. The results indicated that LVEDD, E peak, E/e⁣′, GLS, GWI, GCW, and GWE were critical factors for the LVEF improvement of patients (*p* < 0.05) (Table 4) in univariate logistic regression analysis. There were no significant differences in other variables between improved and nonimproved LVEF patients.

We also employed multivariate logistic regression analysis using GCW (Table 5), GWI (Table 6), and GWE (Table 7) as individual parameters because GWI, GCW, and GWE had strong colinearity to MW in improved LVEF patients. The results confirmed that GCW and GWI were two independent predictive factors (*p* < 0.05) (Tables 5 and 6) for LVEF improvement in patients with HF.

### 3.4. Accurate Analysis of Independent Predictive Factors in Patients with the Improved LVEF Group

**Figure 3:**
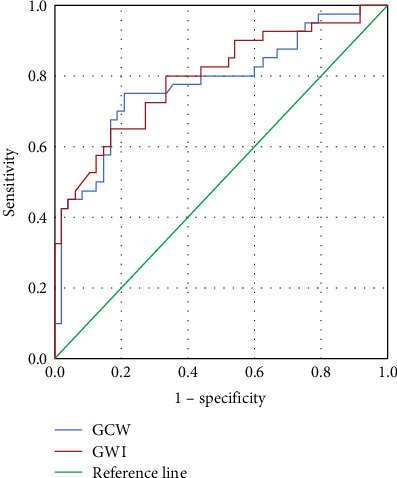
The ROC curve analysis of GCW and GWI. The figure shows the AUC of GCW and GWI by ROC curve. AUC, area under the curve; ROC, receiver operating characteristic; GCW, global constructive work; GWI, global myocardial work index.

Since GCW and GWI were considered as two independent factors for predicting the LVEF improvement of patients with HF, we further evaluated their predictive accuracy using the ROC curve (Figure 3) and calculated the AUC value (Table 8). When GWI was a predictive variable and 741 mmHg% was a cut-off value, the AUC was 0.796, and the 95% confidence interval (CI) was 0.701–0.891. This cut-off value had significantly obtained 65% sensitivity and 83.3% specificity for predicting improved versus nonimproved LVEF patients with HF (*p* < 0.001) (Table 8). Similarly, if GCW was a variable, the AUC was 0.779, and the 95% CI was 0.679–0.880. It got 75% sensitivity and 79.2% specificity at 973.5 mmHg% as the cut-off value for distinguishing improved versus nonimproved LVEF (*p* < 0.001). These data confirmed that GWI and GCW are two critical factors for predicting LVEF improvement of patients with HF.

## 4. Discussion

This study explored the predictive factors for LVEF improvement of patients with HF. We employed a sensitive and reliable PSL technique to measure MW parameters in 88 patients with LVEF ≤ 40% at admission. The results showed that the LVEF of 40 patients (45.5%) had obtained improvement after taking medicine for 6–12 months. Univariate logistic regression analysis indicated that GLS, GWI, GCW, and GWE were crucial factors of LVEF improvement in patients with HF. Multivariate logistic confirmed that GWI and GCW were two determining factors for predicting LVEF improvement in patients with HF. The ROC curve and the AUC calculation demonstrated that GWI and GCW had high sensitivity and specificity at a given cut-off value for the prediction of LVEF improvement in patients with HF.

To narrow down critical predictors for the outcomes of advanced HF, we evaluated differences of basic medical variables and echocardiography measurements between improved and nonimproved LVEF patients at admission and after therapies. The results showed that LVEF, LVEDD, IVST, E peak, GLS, GWI, GCW, and GWE were dramatically different between the two groups. LVEDD, E peak, and E/e⁣′ in patients of the improved group were lower than that in the nonimproved group. Patients with long-term, recurrent HF have severe myocardial cell damage and changes in the left ventricular geometry and function, manifested as increased LVEDD and decreased LVEF [28, 29]. At the time of admission, the two group patients had decreased systolic function and various degrees of diastolic function impairment. If patients had lower LVEF and higher E/e⁣′ ratio, the prognosis was worse [30]. Patients with improved LVEF have higher GLS, which can better reflect subtle changes in the subclinical stages of the diseases, including early myocardial damage when LVEF has not yet been impaired. Therefore, GLS is a more sensitive and accurate method than LVEF detection in predicting the adverse prognosis of HF [31, 32]. In addition, this study shows that the use of anti-HF drugs may not have positive effects on the improvement of LVEF in HFrEF patients, considering the treatment benefits of patients are in a dose-dependent and time-dependent manner [33]. The present data show that there were no significant differences in age, sex, HR, BMI, NYHA class, medical history, platelet count, Hb, HbA1c, and biochemical markers of the two groups of patients (Table 1). In existing HF guidelines and randomized clinical trials (RCTs) about HF, age and sex are not determining factors for predicting the outcomes of HF [34]. One report demonstrated that peripartum cardiomyopathy was associated with sex [35]. However, this cohort study did not include patients with peripartum cardiomyopathy. These results confirm that echocardiography parameters have great benefits for predicting the outcomes of advanced HF. In contrast, age, sex, HR, platelet, Hb, BMI, and biochemical markers are not determining factors.

Suga [36] in 1979 firstly proposed that the area of PSL may stand for MW and oxygen consumption of the LV, which reflect the LV systolic function. Takaoka et al. [37] in 1992 confirmed this concept in laboratory experiments. Russell et al. [8, 9] have successfully obtained MW parameters by noninvasive PSL technique in clinical practice in 2012. Since this report, the PSL technique was quickly applied to measure MW in patients with coronary heart disease, HTN, cardiomyopathy, and so on [38–40]. Recently, PSL was also utilized to assess HF improvement at the late stages from various diseases [26, 41, 42]. The study indicated that PSL detected significantly reduced MWIs at 3 and 6 months after chemotherapies in patients with breast cancer, but normal LVEF [23], indicating PSL is a more sensitive technique for monitoring heart functions. Indeed, the present study revealed that LVEDD, E peak, and GLS of improved LVEF patients were significantly lower than that in nonimproved LVEF patients. In contrast, GWI, GCW, and GWE in the improved group were dramatically higher than those in the nonimproved group. Univariate and multivariate logistic regression analyses confirmed that GWI and GCW were two critical factors for predicting the improvement of HFrEF patients. These results were consistent with previous reports [18, 43]. Hedwig et al. [43] used a GWI ≥ 455 mmHg% and a GCW ≥ 530 mmHg% as cut-off values and then compared the prognosis of HFrEF patients by the Kaplan–Meier curve. The results revealed that the survival time of HFrEF patients with less than these cut-off values was significantly shorter than that of HFrEF patients with greater than these cut-off values. Current results further support that GWI and GCW were two determining predictors for the outcomes of HFrEF patients. Our cut-off values for GWI and GCW were 741 mmHg% and 973.5 mmHg%, respectively. It was higher than the report provided by Hedwig et al. [43], which may be different from human genetic backgrounds, the underlying disease, and measurement equipments. Especially, many studies indicated that GCW was advantageous over other MW parameters in reflecting comprehensive assessment, global perspective, clinical relevance, and sensitivity to changes for myocardial functions [14–16]. The present data confirmed that GCW is a key indicator for the improvement of LVEF in patients with HF. Altogether, our study confirmed that PSL is an excellent technique for measuring MW parameters. It can be used to predict the outcomes of HFrEF patients.

In clinical study, some patients would not receive optimal medical treatments because of many reasons, including intolerable drug side effects, low blood pressure, and so on. There were no significant differences between the two groups in the number of patients treated with medications by statistical analysis. This result showed that PSL can help us to identify patients who may benefit in the short term from treatment for HF.

## 5. Limitations

This study has a few limitations. Firstly, the results were from a single medical center and may give rise to selection bias. Secondly, a small sample size may not reflect general patient conditions. Thirdly, MW data was calculated from echocardiography images. Poor image quality may affect results evaluation. Fourthly, all data were from clinical observation, no mechanism study. In addition, MW parameters were counted based on GLS analysis, and bias may be generated from separated linear regression analysis. Finally, we compared HF improvement after 6–12 months of medicine use. Therefore, we plan to combine more hospital, expand sample size, and explore the mechanisms of MW changes of patients with HF in the future study.

Above results indicated that PSL is a very powerful technique for predicting and evaluating the improvement of LVEF in patients with HF. Therefore, PSL will be applied to identify early subclinical decreased left ventricular myocardial function and the diagnosis, evaluation, and prognosis of HFrEF patients in the future.

## 6. Conclusions

PSL is a sensitive and reliable technique for collecting MW parameters. GWI, GCW, and GWE were strongly associated with the improvement of HFrEF patients. GWI and GCW were two critical factors for predicting the outcomes of HFrEF patients.

## Figures and Tables

**Table 1:** The clinical characteristics of patients.

**Variable**	**LVEF improved (** **n** = 40**)**	**LVEF nonimproved (** **n** = 48**)**	**p**
Age (years)	61.18 ± 13.80	60.71 ± 13.24	0.8721
Sex, *n* (%)			0.5380
M	25 (62.5)	33 (68.8)	
F	15 (37.5)	15 (31.3)	
BW (kg)	70.00 (61.00, 75.00)	71.25 (63.75, 80.00)	0.3396
BMI	25.24 (23.27, 27.51)	25.37 (23.34, 28.89)	0.5242
HR (bpm)	81.50 (72.50, 93.00)	87.00 (77.50, 93.00)	0.4529
Smoking, *n* (%)	12 (30.0)	20 (41.7)	0.2573
Drinking, *n* (%)	7 (17.5)	16 (33.3)	0.092
NYHA class at admission, *n* (%)			0.8208
I	1 (2.5)		
II	3 (7.5)	5 (10.4)	
III	12 (30.0)	13 (27.1)	
IV	24 (60.0)	30 (62.5)	
DM, *n* (%)	14 (35.0)	22 (45.8)	0.3034
HTN, *n* (%)	26 (65.0)	28 (58.3)	0.5225
CAD, *n* (%)			0.3563
No	30 (75.0)	30 (62.5)	
OMI	10 (25.0)	17 (35.4)	
AMI		1 (2.1)	
Arrhythmia, *n* (%)	7 (17.5)	13 (27.1)	0.2854
Hyperlipidaemia, *n* (%)	3 (7.5)	7 (14.6)	0.3364
	7 (17.5)	16 (33.3)	0.092
SBP (mmHg)	125.70 ± 21.89	119.94 ± 15.95	0.1575
DBP (mmHg)	79.00 (70.00, 88.50)	80.00 (70.00, 90.00)	0.6799
Serum glucose (*μ*mol/L)	5.17 (4.62, 7.16)	6.06 (5.05, 7.62)	0.134
Serum potassium (mmol/L)	4.21 ± 0.47	4.30 ± 0.41	0.331
Serum albumin (g/L)	39.70 (38.05, 41.95)	39.90 (35.10, 42.70)	0.878
NT-proBNP (pg/mL)	4821.20 (780.25, 12448.50)	4748.00 (1639.50, 10776.10)	0.750
LDL (mmol/L)	2.75 ± 0.802	2.56 ± 0.91	0.312
CK-MB (ng/mL)	1.73 (0.98, 2.85)	1.49 (1.00, 2.30)	0.4506
Creatinine (*μ*mol/L)	83.20 (58.95, 124.85)	82.90 (68.60, 101.10)	0.9766
Hb (g/L)	144.50 (125.50, 154.50)	146.00 (128.00, 161.50)	0.2027
Platelet (1 × 10^9^/L)	240.58 ± 62.26	240.25 ± 69.53	0.982
HbA1c (%)	6.57 ± 1.46	6.85 ± 1.78	0.421
Hospitalization time (days)	7.28 ± 2.72	7.06 ± 2.23	0.688

Abbreviations: AMI, acute myocardial infarction; BMI, body mass index; BW, body weight; CAD, coronary artery disease; CK-MB, creatine kinase isoenzyme; DBP, diastolic blood pressure; DM, diabetes mellitus; F, female; Hb, hemoglobin; HF, heart failure; HR, heart rate; HTN, hypertension; LDL, low-density lipoprotein; M, male; *n*, case number; NT-proBNP, N-terminal protein hormone B-natriuretic peptide; NYHA, New York Heart Association; OMI, old myocardial infarction; SBP, systolic blood pressure.

**Table 2:** General echocardiography parameters comparison in LVEF improved and nonimproved patients.

**Variable**	**LVEF improved (** **n** = 40**)**	**LVEF nonimproved (** **n** = 48**)**	**p**
LVEF (%) at admission	35.00 (26.00, 39.75)	30.00 (25.00, 36.00)	**0.038**
LVEDD (mm)	56.63 ± 5.46	61.10 ± 7.76	**0.0029**
IVST (mm)	10.00 (9.30–10.80)	9.60 (8.35–10.70)	**0.0362**
LVPWT (mm)	9.87 ± 1.20	9.67 ± 1.26	0.4569
E peak (cm/s)	70.00 (49.00, 84.50)	78.00 (60.00–110.00)	**0.0260**
E/e⁣′	15.09 ± 5.841	19.31 ± 9.358	0.054
GLS (%)	−8.60 ± 2.96	−6.13 ± 2.15	**< 0.0001**
GWI (mmHg%)	859.48 ± 311.33	542.27 ± 200.56	**< 0.0001**
GCW (mmHg%)	1152.75 ± 349.19	816.83 ± 248.54	**< 0.0001**
GWE (mmHg%)	79.95 ± 9.99	72.58 ± 9.17	**0.0005**
GWW (mmHg%)	200.00 (151.50–350.00)	248.50 (177.50–344.50)	0.1655

*Note:* All *p* values < 005 in the table are marked in bold, indicating the statistical significance of the differences.

Abbreviations: GCW, global myocardial constructive work; GLS, global longitudinal strain; GWE, global myocardial work efficiency; GWI, global myocardial work index; GWW, global wasted work; IVST, interventricular septum thickness; LVEDD, left ventricle; LVEF, left ventricle ejection fraction; LVPWT, left ventricle posterior wall thickness.

**Table 3:** The correlation between myocardial work and the LVEF improved patients.

**Variable**	**r**	**LVEF value change**
		**p**
GWI (mmHg%)	0.57997	**< 0.0001**
GCW (mmHg%)	0.54917	**< 0.0001**
GWW (mmHg%)	−0.08959	0.4065
GWE (mmHg%)	0.42794	**< 0.0001**

*Note:* All *p* values < 005 in the table are marked in bold, indicating the statistical significance of the differences.

Abbreviations: GCW, global myocardial constructive work; GWE, global myocardial work efficiency; GWI, global myocardial work index; GWW, global wasted work; LVEF, left ventricular ejection fraction.

**Table 4:** Univariate logistic regression analysis in LVEF improved patients.

**Variable**	**Β** ** value**	**SD**	**W** **a** **l** **d** **χ** ^2^ ** value**	**p**	**OR (95% CI)**
Age (years)	−0.001	0.016	0.005	0.942	0.999 (0.997–2.032)
Sex	−0.189	0.457	0.171	0.679	0.828 (0.338–2.028)
Hb (g/L)	0.018	0.010	3.365	0.067	1.018 (0.999–1.038)
*β* Receptor blocker	−0.879	0.514	2.924	0.087	0.415 (0.152–1.137)
Drink history	−0.857	0.517	2.755	0.097	0.424 (0.154–1.168)
TnI (pg/mL)	**<** −0.001	**<** 0.001	0.023	0.880	1.000 (0.999–1.000)
CK-MB (ng/mL)	−0.291	0.164	3.150	0.076	0.748 (0.542–1.031)
NT-proBNP (pg/mL)	−4.715	**<** 0.001	0.047	0.829	1.000 (1.000–1.000)
LVEF at admission	−0.058	0.032	3.346	0.067	0.943 (0.886–1.004)
IVST (mm)	−0.267	0.138	3.735	0.053	0.766 (0.584–1.004)
LVEDD (mm)	0.101	0.036	7.877	**0.005**	1.107 (1.031–1.188)
LVPWT (mm)	−0.133	0.177	0.564	0.453	0.876 (0.619–1.239)
E peak (cm/s)	0.021	0.009	5.913	**0.015**	1.021 (1.004–1.038)
E/e'	0.070	0.030	5.430	**0.020**	1.073 (1.011–1.138)
GLS(%)	0.397	0.108	13.528	**< 0.001**	1.487 (1.203–1.836)
GWI (mmHg%)	−0.005	0.001	17.733	**< 0.001**	0.995 (0.993–0.997)
GCW (mmHg%)	−0.004	< 0.001	16.913	**< 0.001**	0.996 (0.994–0.998)
GWW (mmHg%)	0.001	0.002	0.682	0.409	1.001 (0.998–1.004)
GWE (mmHg%)	−0.081	0.025	10.346	**0.001**	0.922 (0.878–0.969)
HbA1c (%)	−0.284	0.086	0.35	0.421	0.808 (0.415–0.984)
Platelet (1 × 10^9^/L)	−0.325	0.463	14.2	0.982	0.023 (0.403–0.971)
Hospitalization time (days)	−0.213	0.176	0.527	0.688	0.403 (−1 to 26 to 0.835)

*Note:* All *p* values < 005 in the table are marked in bold, indicating the statistical significance of the differences.

Abbreviations: CI, confidence intervals; CK-MB, creatine kinase isoenzyme; GCW, global myocardial constructive work; GLS, global longitudinal strain; GWE, global myocardial work efficiency; GWI, global myocardial work index; GWW, global wasted work; Hb, hemoglobin; IVST, interventricular septum thickness; LVEDD, left ventricle; LVEF, left ventricle ejection fraction; LVPWT, left ventricle posterior wall thickness; NT-proBNP, N-terminal probrain natriuretic peptide; SD, standard deviation; TnI; troponin I.

**Table 5:** Multivariate logistic regression analysis of GCW for the LVEF improvement of patients.

**Variable**	**β** ** value**	**SD**	**χ** ^2^	**p**	**OR (95% CI)**
E peak (cm/s)	0.003	0.013	0.050	0.823	1.003 (0.978, 1.029)
E/e'	0.009	0.045	0.036	0.850	1.009 (0.924, 1.101)
LVEDD (mm)	0.065	0.041	2.575	0.109	1.068 (0.986, 1.156)
GLS (%)	0.077	0.155	0.250	0.617	1.080 (0.798, 1.463)
GCW (mmHg%)	−0.003	0.001	4.193	**0.041**	0.997 (0.994, 1.000)

*Note:* All *p* values < 005 in the table are marked in bold, indicating the statistical significance of the differences.

Abbreviations: CI, confidence intervals; GCW, global myocardial constructive work; GLS, global longitudinal strain; LVEDD, left ventricular end-diastolic diameter; LVEF, left ventricular ejection fraction; OR, odds ratio; SD, standard deviation.

**Table 6:** Multivariate logistic regression analysis of GWI for the LVEF improvement of patients.

**Variable**	**β** ** value**	**SD**	**χ** ^2^	**p**	**OR (95% CI)**
E peak (cm/s)	0.006	0.013	0.212	0.645	1.006 (0.981, 1.032)
E/e⁣′	0.007	0.046	0.022	0.883	1.007 (0.920, 1.102)
LVEDD (mm)	0.060	0.042	2.017	0.156	1.061 (0.978, 1.152)
GLS (%)	−0.090	0.189	0.226	0.634	0.914 (0.632, 1.323)
GWI (mmHg%)	−0.005	0.002	5.928	**0.015**	0.995 (0.991, 0.999)

*Note:* All *p* values < 005 in the table are marked in bold, indicating the statistical significance of the differences.

Abbreviations: CI, confidence intervals; GLS, global longitudinal strain; GWI, global myocardial index; LVEDD, left ventricular end-diastolic diameter; LVEF, left ventricular ejection fraction; OR, odds ratio; SD, standard deviation.

**Table 7:** Multivariate logistic regression analysis of GWE for the LVEF improvement of patients.

**Variable**	**β** ** value**	**SD**	**χ** ^2^	**p**	**OR (95% CI)**
E peak (cm/s)	0.012	0.013	0.875	0.350	1.012 (0.987, 1.037)
E/e⁣′	0.006	0.046	0.017	0.896	1.006 (0.920, 1.100)
LVEDD (mm)	0.065	0.040	2.620	0.106	1.067 (0.986, 1.155)
GLS (%)	0.257	0.156	2.730	0.098	1.293 (0.953, 1.754)
GWE (mmHg%)	−0.019	0.037	0.264	0.607	0.981 (0.912, 1.055)

Abbreviations: CI, confidence interval; GLS, global longitudinal strain; GWE, global myocardial work efficiency; LVEDD, left ventricular end-diastolic diameter; LVEF, left ventricular ejection fraction; OR, odds ratio; SD, standard deviation.

**Table 8:** AUC comparison of independent predictive factors for LVEF-improved patients.

**Variable**	**Cut-off**	**Sensitivity**	**Specificity**	**Youden index**	**AUC**	**95% CI**	**p** ** value**
GWI (mmHg%)	741.000	0.650	0.833	0.483	0.796	0.701–0.891	< **0.001**
GCW (mmHg%)	973.500	0.750	0.792	0.542	0.779	0.679–0.880	< **0.001**

*Note:* All *p* values < 005 in the table are marked in bold, indicating the statistical significance of the differences.

Abbreviations: AUC, area under curve; CI, confidence intervals; GCW, global myocardial constructive work; GWI, global myocardial work index; LVEF, left ventricular ejection.

## Data Availability

The datasets used and/or analyzed during the current study are available from the corresponding author on reasonable request.

## References

[1] Punnoose L. R., Givertz M. M., Lewis E. F., Pratibhu P., Stevenson L. W., Desai A. S. (2011). Heart failure with recovered ejection fraction: a distinct clinical entity. *Journal of Cardiac Failure*.

[2] Heidenreich P. A., Bozkurt B., Aguilar D. (2022). 2022 AHA/ACC/HFSA guideline for the management of heart failure: executive summary: a report of the American College of Cardiology/American Heart Association joint committee on clinical practice guidelines. *Journal of the American College of Cardiology*.

[3] Heart Failure Group of Chinese Society of Cardiology of Chinese Medical Association (2018). Chinese guidelines for the diagnosis and treatment of heart failure 2018. *Zhonghua Xin Xue Guan Bing Za Zhi*.

[4] Hamabe L., Mandour A. S., Shimada K. (2021). Role of two-dimensional speckle-tracking echocardiography in early detection of left ventricular dysfunction in dogs. *Animals*.

[5] Liu Q., Chen L., Liu X. (2023). Evaluation of left ventricular myocardial work in patients with hyperthyroidism with different heart rates with noninvasive pressure-strain loop based on two-dimensional speck tracking imaging. *Quantitative Imaging in Medicine and Surgery*.

[6] Tang S., Li H., Song L., Zhou Y. (2023). Echocardiographic study of left ventricular pressure-strain loop in evaluating changes in left ventricular myocardial work in breast cancer patients after chemotherapy. *International Heart Journal*.

[7] Cao Y., Zhang H., Li S. (2023). Correlation analysis between myocardial work indices and liver function classification in patients with hepatitis B cirrhosis: a study with non-invasive left ventricular pressure-strain loop. *Frontiers in Cardiovascular Medicine*.

[8] Russell K., Eriksen M., Aaberge L. (2012). A novel clinical method for quantification of regional left ventricular pressure-strain loop area: a non-invasive index of myocardial work. *European Heart Journal*.

[9] Russell K., Eriksen M., Aaberge L. (2013). Assessment of wasted myocardial work: a novel method to quantify energy loss due to uncoordinated left ventricular contractions. *American Journal of Physiology. Heart and Circulatory Physiology*.

[10] Abawi D., Rinaldi T., Faragli A. (2022). The non-invasive assessment of myocardial work by pressure-strain analysis: clinical applications. *Heart Failure Reviews*.

[11] Manganaro R., Marchetta S., Dulgheru R. (2019). Echocardiographic reference ranges for normal non-invasive myocardial work indices: results from the EACVI NORRE study. *Imaging*.

[12] Hedwig F., Soltani S., Stein J. (2020). Global work index correlates with established prognostic parameters of heart failure. *Echocardiography*.

[13] Moya A., Buytaert D., Penicka M., Bartunek J., Vanderheyden M. (2023). State-of-the-art: noninvasive assessment of left ventricular function through myocardial work. *Journal of the American Society of Echocardiography*.

[14] Miric D., Bakovic D., Zanchi J., Bradaric Slujo A., Lozo M., Borovac J. A. (2024). Myocardial work in patients with heart failure and ischemic cardiomyopathy according to the mode of coronary revascularization. *Hellenic Journal of Cardiology*.

[15] Ran H., Yao Y., Wan L. (2023). Characterizing stenosis severity of coronary heart disease by myocardial work measurement in patients with preserved ejection fraction. *Quantitative Imaging in Medicine and Surgery*.

[16] Sakhnova T. A., Dobrovolskaya S. V., Blinova E. V., Uskach T. M., Saidova M. A. (2023). Relationship between parameters of myocardial electrical remodeling and parameters of contractile function, deformation and myocardium work in patients with chronic heart failure with low left ventricular ejection fraction and atrial fibrillation. *Kardiologiia*.

[17] Tadic M., Cuspidi C., Pencic B., Grassi G., Celic V. (2020). Myocardial work in hypertensive patients with and without diabetes: an echocardiographic study. *Journal of Clinical Hypertension (Greenwich, Conn.)*.

[18] Li Y., Zheng Q., Cui C. (2022). Application value of myocardial work technology by non-invasive echocardiography in evaluating left ventricular function in patients with chronic heart failure. *Quantitative Imaging in Medicine and Surgery*.

[19] Edwards N. F. A., Scalia G. M., Shiino K. (2019). Global myocardial work is superior to global longitudinal strain to predict significant coronary artery disease in patients with normal left ventricular function and wall motion. *Journal of the American Society of Echocardiography*.

[20] Hubert A., Le Rolle V., Leclercq C. (2018). Estimation of myocardial work from pressure-strain loops analysis: an experimental evaluation. *European Heart Journal Cardiovascular Imaging*.

[21] Chan J., Edwards N. F. A., Khandheria B. K. (2019). A new approach to assess myocardial work by non-invasive left ventricular pressure-strain relations in hypertension and dilated cardiomyopathy. *European Heart Journal Cardiovascular Imaging*.

[22] Di Lisi D., Manno G., Novo G. (2021). Subclinical cardiotoxicity: the emerging role of myocardial work and other imaging techniques. *Current Problems in Cardiology*.

[23] Di Lisi D., Manno G., Madaudo C. (2023). Chemotherapy-related cardiac dysfunction: the usefulness of myocardial work indices. *The International Journal of Cardiovascular Imaging*.

[24] Pugliese N. R., Fabiani I., Santini C. (2019). Value of combined cardiopulmonary and echocardiography stress test to characterize the haemodynamic and metabolic responses of patients with heart failure and mid-range ejection fraction. *European Heart Journal Cardiovascular Imaging*.

[25] Paolisso P., Gallinoro E., Mileva N. (2022). Performance of non-invasive myocardial work to predict the first hospitalization for de novo heart failure with preserved ejection fraction. *ESC Heart Failure*.

[26] Bouali Y., Donal E., Gallard A. (2020). Prognostic usefulness of myocardial work in patients with heart failure and reduced ejection fraction treated by sacubitril/valsartan. *The American Journal of Cardiology*.

[27] Wilcox J. E., Fang J. C., Margulies K. B., Mann D. L. (2020). Heart failure with recovered left ventricular ejection fraction. *Journal of the American College of Cardiology*.

[28] Soylu K., Cerik I. B., Aksan G., Nar G., Meric M. (2020). Evaluation of ivabradine in left ventricular dyssynchrony and reverse remodeling in patients with chronic heart failure. *Journal of Arrhythmia*.

[29] Wang Y., Zhou R., Lu C., Chen Q., Xu T., Li D. (2019). Effects of the angiotensin-receptor neprilysin inhibitor on cardiac reverse remodeling: meta-analysis. *Journal of the American Heart Association*.

[30] Xiaoli W., Junhua Y., Bingyuan Z., Caiming Z. (2013). Value of echocardiography parameter E/Ea in evaluating prognosis of patients with systolic heart failure. *Paper Read at the 15th National Congress of Cardiology*.

[31] Nikoo M. H., Naeemi R., Moaref A., Attar A. (2020). Global longitudinal strain for prediction of ventricular arrhythmia in patients with heart failure. *ESC Heart Failure*.

[32] Aalen J. M., Donal E., Larsen C. K. (2020). Imaging predictors of response to cardiac resynchronization therapy: left ventricular work asymmetry by echocardiography and septal viability by cardiac magnetic resonance. *European Heart Journal*.

[33] Martens P., Beliën H., Dupont M., Vandervoort P., Mullens W. (2018). The reverse remodeling response to sacubitril/valsartan therapy in heart failure with reduced ejection fraction. *Cardiovascular Therapeutics*.

[34] McDonagh T. A., Metra M., Adamo M. (2023). 2023 focused update of the 2021 ESC guidelines for the diagnosis and treatment of acute and chronic heart failure. *European Heart Journal*.

[35] Chinese Society of Cardiology, Chinese Medical Association (2024). Chinese guidelines for the diagnosis and treatment of heart failure 2024. *Zhonghua Xin Xue Guan Bing Za Zhi*.

[36] Suga H. (1979). Total mechanical energy of a ventricle model and cardiac oxygen consumption. *The American Journal of Physiology*.

[37] Takaoka H., Takeuchi M., Odake M., Yokoyama M. (1992). Assessment of myocardial oxygen consumption (Vo2) and systolic pressure-volume area (PVA) in human hearts. *European Heart Journal*.

[38] Wang R. R., Tian T., Li S. Q., Leng X. P., Tian J. W. (2021). Assessment of left ventricular global myocardial work in patients with different degrees of coronary artery stenosis by pressure-strain loops analysis. *Ultrasound in Medicine & Biology*.

[39] Ding J., Sun H. G., Liu J., Wu D. (2022). Assessment of left ventricular myocardial work done by noninvasive pressure-strain loop technique in patients with essential hypertension. *Annals of Noninvasive Electrocardiology*.

[40] Cui C., Li Y., Liu Y. (2021). Association between echocardiographic non-invasive myocardial work indices and myocardial fibrosis in patients with dilated cardiomyopathy. *Frontiers in Cardiovascular Medicine*.

[41] Tomoaia R., Beyer R. S., Zdrenghea D. (2021). Global work index by non-invasive pressure-strain loops: a novel parameter to assess left ventricular performance in the early stages of heart failure with preserved or mid-range ejection fraction after acute myocardial infarction. *Medical Ultrasonography*.

[42] Nakamura M., Sunagawa O., Hokama R. (2016). A case of refractory heart failure in Becker muscular dystrophy improved with corticosteroid therapy. *International Heart Journal*.

[43] Hedwig F., Nemchyna O., Stein J. (2021). Myocardial work assessment for the prediction of prognosis in advanced heart failure. *Frontiers in Cardiovascular Medicine*.

