# Supplementary material for: Application of the Left Ventricular Pressure–Strain Loop Technique in Monitoring Improvement Factors of Patients With Heart Failure Reduced Ejection Fraction
Source: Cardiovasc Ther. 2024 Dec 17;2024:5562513. doi: 10.1155/cdr/5562513 (PMC11668549; doi:10.1155/cdr/5562513)
Supplement: Supporting Information — Additional supporting information can be found online in the Supporting Information section. Table S1: effects of medicine use in LVEF patients. [file 5562513.f1.docx]

**Supplementary Table 1 Effects of Medicinesuse in patients LVEF**

| Category | Drug Name | Dose ,route | Precaution | LVEF  Improved (n=40) | LVEF  Non-improved(n=48) | P value |
| --- | --- | --- | --- | --- | --- | --- |
| ARNI, n(%)  ACEI,n(%)  ARB,n(%) | Entresto  0  1  Enalapril  0  1  Valsartan  0  1 | 25-200mg  oral, bid  5-20mg  Oral,qd  80-160mg  Oral,qd | No use for SBP<90mmHg  Or K+ >5.5mmol/L  Same above  Same above | 7（17.5）  33（82.5）  39（97.5）  1（2.5）  39（97.5）  1（2.5） | 15（31.2）  33（68.8）  47(97.9)  1(2.1)  44（91.7）  4（8.3） | 0.138  0.475 |
| β receptor blocker, n(%) | Metoprolol succinate  0  1  Bisoprolol  0  1 | 47.5-190mg  Oral,qd  2.5-5mg  Oral ,qd | No use for SBP<90mmHg  or HR <60 BPM  same above | 25（62.5）  15（37.5）  28（70.0）  12（30.0） | 28（58.3）  20（41.7）  28（58.3）  20（41.7） | 0.691  0.257 |
| MRA, n(%) | Spironolactone  0  1 | 20mg  Oral, qd | No use forK+ >5.5mmol/L | 16(40.0)  24(60.0) | 22(45.8)  26(54.2) | 0.582 |
| SGLT-2 receptor inhibitor, n(%) | Dapagliflozin  0  1  Empagliflozin  0  1 | 10mg  Oral,qd  10mg  Oral, qd | ----  ---- | 29(72.5)  11(27.5)  35（87.5）  5（12.5） | 33(58.3) 15(41.7)  43（89.6）  5（10.4） | 0.701  0.759 |
| Diuretics | Furosemide  0  1  Torasemide  0  1 | 20-100mg  IV,qd  20-100mg  IV,qd | No use for SBP<90mmHg  ,monitoring K+ concentration  Same as above | 20(50.0)  20(50.0)  28（70.0）  12（30.0） | 25(52.1)  23(47.9)  38（79.2）  10（20.8） | 0.846  0.323 |

LVEF, left ventricle ejection factor; n, case number; 0, no drug use; 1 drug use; ARNI,angiotensin receptor /neprilysin inhibitor;ACEIs, angiotensin-converting enzyme inhibitors; ARB,angiotensin receptor blockers;MRA, aldosterone receptor antagonist; SBP, systolic blood pressure; K+, serum potassium;IV, in vein.
